# A Multi-Node Magnetic Positioning System with a Distributed Data Acquisition Architecture

**DOI:** 10.3390/s20216210

**Published:** 2020-10-30

**Authors:** Francesco Santoni, Alessio De Angelis, Antonio Moschitta, Paolo Carbone

**Affiliations:** Department of Engineering, University of Perugia, 06125 Perugia, Italy; alessio.deangelis@unipg.it (A.D.A.); antonio.moschitta@unipg.it (A.M.); paolo.carbone@unipg.it (P.C.)

**Keywords:** positioning, tracking, magnetic fields, distance measurement, resonators

## Abstract

We present a short-range magnetic positioning system that can track in real-time both the position and attitude (i.e., the orientation of the principal axes of an object in space) of up to six moving nodes. Moving nodes are small solenoids coupled with a capacitor (resonant circuit) and supplied with an oscillating voltage. Active moving nodes are detected by measuring the voltage that they induce on a three-dimensional matrix of passive coils. Data on each receiving coil are acquired simultaneously by a distributed data-acquisition architecture. Then, they are sent to a computer that calculates the position and attitude of each moving node. The entire process is run in real-time: the system can perform 62 position and attitude measurements per second when tracking six nodes simultaneously and up to 124 measurements per second when tracking one node only. Different active nodes are identified using a frequency-division multiple access technique. The position and angular resolution of the system have been experimentally estimated by tracking active nodes along a reference trajectory traced by a robotic arm. The factors limiting the viability of upscaling the system with more than six active nodes are discussed.

## 1. Introduction

Magnetic positioning systems (MPS) are widely investigated as a suitable solution for applications needing localization in space of magnetic transmitters aptly mounted on objects or people [1]. The growing field of the Internet of Things (IoT) provides many application scenarios for localization systems [2], such as wireless sensor networks [3,4], mobile robots [5], and location-based services [6]. The main advantage of using localization systems based on a magnetic field is that they do not need line-of-sight conditions to work. For this reason, an extensively investigated application is the tracking of capsule probes inside the human body, i.e., wireless capsule endoscopy [7,8]. Non-line-of-sight conditions also occur in many indoor applications, whenever the localization of a magnetic transmitter inside buildings is required [9,10,11].

Short-range MPSs [12,13] can be used to accurately navigate surgical instruments [13,14], to investigate motor symptoms of diseases such as Parkinson’s disease [15,16], to provide a human–machine interface such as in data gloves [17,18]. In the latter case, magnetic transmitters and/or sensors are mounted on the fingers in order to track their movements. This latter application would require tracking multiple magnetic nodes simultaneously, a topic that has been investigated in the last few years.

In [19], multiple magnetic markers are localized using Hall sensors. The magnetic field of the markers can be analytically calculated by modeling the markers as elementary magnetic dipoles. Measuring the magnetic field, marker positions are obtained by numerically inverting the model. The real-time simultaneous tracking of up to seven markers is reported. However, different markers cannot be identified, i.e., it is not possible to tell which marker is on each of the measured positions unless this information is known a priori. In [20], magnetic transmitters are coils supplied with a 119-kHz AC current. Signal separation and identification are obtained by modulating the coils with on–off-keying using orthogonal code sequences, an approach called Code Division Multiple Access (CDMA). A CDMA technique using Chirp Spread Spectrum signals has been recently investigated for an MPS with twenty magnetic transmitters [21]. In a different approach [22], each magnetic node transmits using a different frequency and signals are separated by Fourier analysis. This technique is called Frequency Division Multiple Access (FDMA).

Instead of using Hall sensors, in many MPSs, a time-varying magnetic field is measured by probing the voltage induced on passive coils, in accordance with Faraday’s induction law [11,12,14,21,22,23,24,25]. Receiving coils are coupled with capacitors to form resonant inductor-capacitor (LC) circuits. Tuning transmitter frequencies on the resonance band of the receivers has a double advantage: first, the induced voltage is increased without increasing the absorbed power of the transmitter; second, the LC circuit acts as a filter, selecting only frequencies of interest within the resonance band, filtering wide- and narrow-band noise components.

In this work, we present a short-range MPS, including its performance results. Resonant coils are used as receivers. Also, the transmitters are small LC circuits supplied with an alternating current, thus generating a time-varying magnetic field. Multi-node tracking is based on the FDMA technique. The position and attitude of each node are measured (five degrees of freedom (DOFs) for each node; the attitude is defined as the direction of the solenoid axis). A novel distributed data-acquisition (DAQ) architecture has been implemented for fast performance. The system can perform up to 124 measurements per second of a single magnetic node and 62 measurements per second of six nodes. The position and attitude accuracy are a few millimiters and a few degrees, respectively. The applicability of the present system for measuring hand tremor associated with Parkinson’s disease has already been proved by tracking a single node [16]. Multi-node tracking can be exploited in data gloves equipped with magnetic sensors, e.g., to assess hand kinematics and functioning [18,26], to drive industrial equipments by gesture recognition [27], or to devise automatic sign-language recognition systems [28].

## 2. Description of the System

### 2.1. Measurement Principle

Our MPS is devised to track small solenoids (TX) supplied with an oscillating current. When traversed by an electric current, each TX is equivalent to a magnetic dipole, according with Ampere’s equivalence principle. Since an alternating current is supplied, each TX produces a varying magnetic field. This field induces on passive solenoids (RX) an electromotive force proportional to the time derivative of the flux of the magnetic field that crosses each RX coil surface, in accordance with Faraday’s principle.

### 2.2. System Features

The system is based on a matrix of RXs mounted at fixed positions. By measuring the signal induced on each RX solenoid and by inverting a simple mathematical model based on the dipole approximation (i.e., each coil is treated like an elementary magnetic dipole), it is possible to extract the position and attitude of each mobile TX node.

The MPS presented in this work is an enhanced realization of the system presented in [12]. It includes 24 RX sensors: 16 sensors are mounted on the xy plane, while 4 sensors are mounted on both the xz and yz planes. On the horizontal plane, sensors are arranged on a regular 4×4 grid, with the distance between sensors being 8 cm. On vertical planes, sensors are positioned on the vertices of a square in which the sides are 8 cm, at the center of both planes. The active volume of the system is a 30×30×30 cm${}^{3}$ box, but since a TX cannot be put too close to an RX because the signal magnitude would be too high, implying saturation problems, the effective active volume should be considered a 25×25×25 cm${}^{3}$ box.

**Figure 1:**
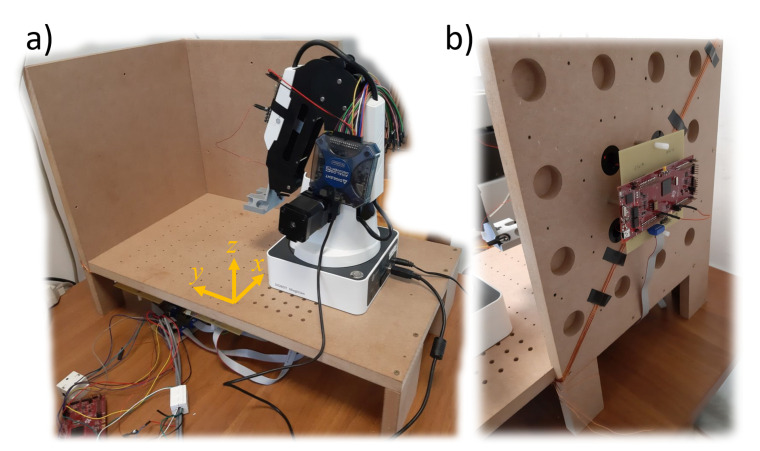
A picture of the system: (**a**) the whole magnetic positioning system with a robotic arm mounted on it. The reference frame is indicated. (**b**) A rear view of the yz plane: the round, carved slots for the receivers are visible; only four receivers are mounted on this plane; signals on the receivers are acquired by a microcontroller unit (the red board in the picture); and the microcontroller is mounted on a custom circuit board (the yellow board) described in Section 2.10. The other four receivers with a microcontroller unit are mounted on the xz plane; sixteen receivers are mounted under the xy plane with four microcontrollers. (The diagonal wire was used to wirelessly power the small solenoid (TX) in a different setup not treated in this paper.).

The supporting structure of the RX matrix is made of wood and has been cut with a numerically controlled milling machine (Figure 1). RX solenoids are mounted into carved slots. The xy plane provides also a support for the robotic arm used to move the TXs along controlled trajectories. A matrix of slots was also carved on the xy plane to secure the robot on fixed positions and to easily place its end effector on fixed grid points as a reference to calibrate the movement of the robotic arm by performing the procedure described in [12].

The system can track up to six TX nodes simultaneously, while the system in [12] could track one node only. In order to discriminate different nodes, each node is supplied with a different voltage frequency, thus implementing the FDMA technique. The received signal on each RX is the sum of several frequency components that can be separated by Fourier analysis.

Signals on each RX are acquired by analog-to-digital converters (ADC). In [12], a single ADC was multiplexed to sample all channels, using a pseudo-simultaneous strategy: accordingly, the sampling frequency $F_{s}$ for each channel was equal to the sampling frequency of the ADC, divided by the number of channels. For the enhanced MPS described in this work, the idea is to use a distributed DAQ architecture completely parallelized [29] to sample all channels simultaneously. The signal on each channel is acquired by an independent ADC. Signal parameter extraction is performed by several microcontroller units (MCU) working in parallel. All extracted parameters are collected by a single MCU and then transferred to a computer that estimates the position and attitude of each TX.

### 2.3. Principle of Operation

The active TX solenoid is treated as an elementary magnetic dipole $m_{tx}$ [22]:

(1) $$m_{tx}=m_{tx}{\hat{n}}_{tx},\;\;\;m_{tx}=N_{tx}S_{tx}I,\;\;\;S_{tx}=\pi r_{tx}^{2}$$

where ${\hat{n}}_{tx}$ is the versor orthogonal to the coil surface and defines what we call the attitude of the TX; $r_{tx}$ and $S_{tx}$ are its radius and coil surface, respectively; $N_{tx}$ is the number of windings; and *I* is the root mean square (RMS) value of the sinusoidal current of frequency $f_{0}$ flowing in the coil, for which the peak value is $I_{0}=I\sqrt{2}$. The RMS magnetic field induced by a TX at the center of the *i*th RX receiver is as follows:

(2) $$B_{rms,i}=\frac{\mu_{0}}{4\pi }\frac{m_{tx}}{d_{i}^{3}}\left[3\left({\hat{n}}_{tx}\cdot {\hat{n}}_{d,i}\right){\hat{n}}_{d,i}-{\hat{n}}_{tx}\right],$$

where $d_{i}$ is the distance between TX and the *i*th RX, and ${\hat{n}}_{d,i}$ is the unit vector associated with $d_{i}=r_{rx,i}-r_{tx}$. By approximating B as being homogeneous over the whole RX coil surface, the RMS voltage induced on the *i*th RX is as follows:

(3) $$V_{rms,i}=2\pi f_{0}N_{rx}S_{rx}B_{rms,i}\cdot {\hat{n}}_{rx,i},$$

where $N_{rx}$ is the number of windings of each RX coil, $S_{rx}$ is its surface, and ${\hat{n}}_{rx,i}$ is its unit vector assuming a left-handed orientation of the coil.

If we collect position and attitude together as a single vector $\theta ={\left[r_{tx}\;{\hat{n}}_{tx}\right]}^{T}$, its value can be estimated by minimizing the following cost function:

(4) $$F\left(\theta \right)=\sum_{i}{\left[{\tilde{V}}_{rms,i}-V_{rms,i}\left(\theta \right)\right]}^{2},$$

where ${\tilde{V}}_{rms,i}$ is the measured voltages on each RX. Accordingly, $\hat{\theta }=\underset{\theta }{arg min}F\left(\theta \right)$.

### 2.4. System Calibration

An accurate knowledge of receiver positions and orientations is necessary for accurate tracking. A further source of error is the uncertainty on $m_{tx}$ [23] and on other quantities such as the RX surface $S_{rx}$ and the effective number of windings $N_{rx}$. Moreover, each RX coil is coupled with parallel capacitance, thus forming a resonant circuit (see Section 2.5) with a voltage gain $G_{Q}\left(\omega \right)$ varying with frequency. The signal on each RX is also amplified before being acquired, using an instrumentation amplifier (INA; see Section 2.10) with gain $G_{ina}$. Hence, the measured voltage is $G_{Q}G_{ina}V_{rms}$, and the uncertainties on $G_{Q}$ and $G_{ina}$ have to be taken into account.

A calibration procedure is thus needed in order to set as accurately as possible all these system parameters. Each active TX is moved along a reference trajectory, i.e., over a known values of $\tilde{\theta }\left(k\right)$, where *k* indicates the time step. Then, the position and attitude of each RX, $\theta_{rx,i}={\left[r_{rx,i}\;{\hat{n}}_{rx,i}\right]}^{T}$, are evaluated by minimizing the cost function:

(5) $$F\left(\theta_{rx,i}\right)=\sum_{k}\sum_{i}{\left[{\tilde{V}}_{rms,i}-V_{rms,i}\left(\tilde{\theta }\left(k\right),\theta_{rx,i},C_{rx,i}\right)\right]}^{2}.$$

In (5), we have explicitly indicated also the calibration constants $C_{rx,i}$ defined in [12] that account for uncertainties on all other parameters listed above. Basically, since all the other parameters are multiplicative factors in the expression of $V_{rms}$, the voltage actually measured is $C_{rx,i}G_{Q}G_{ina}V_{rms}$. By solving the minimization problem, $C_{rx,i}$ is also estimated, thus compensating for all the uncertainties on the system parameters. The calibration procedure is described with full detail in [12]. For both positioning and calibration, the nonlinear minimization problem can be solved using the *concentrated cost function* method introduced in [30], i.e., decomposing the problem into a linear and a reduced nonlinear problem. Once the system has been calibrated, as it is used to track a trajectory, the estimate of $\hat{\theta }\left(k\right)$ is made more accurate and stable by introducing a *Kalman filter* [31,32] based on the *nearly constant velocity model* [32]. The application of the concentrated cost function and Kalman filter to the MPS are illustrated in [12].

### 2.5. Coils and Alternating Voltage Supply

**Figure 2:**
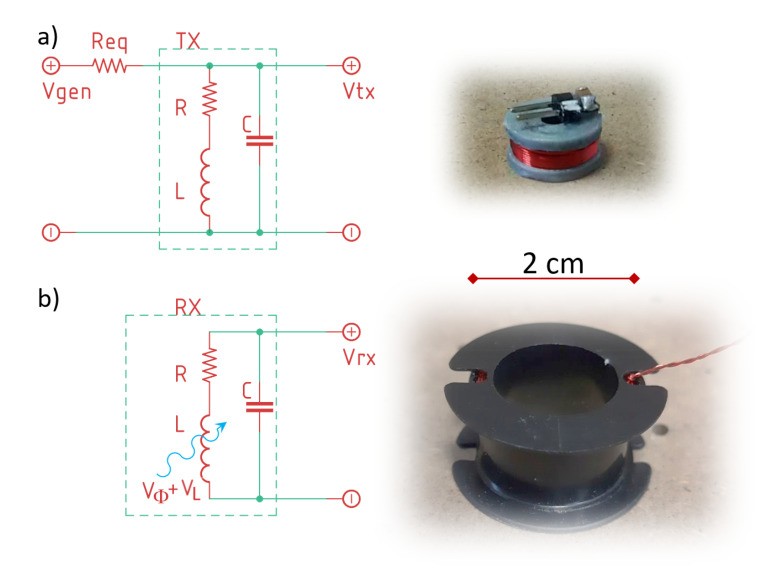
A picture of the TX (**a**) and RX (**b**) solenoids with their circuit model: TX is supplied with voltage $V_{gen}$ from a waveform generator with an equivalent resistance $R_{eq}$. On the RX solenoid, a voltage $V_{\Phi }$ is induced by the external magnetic flux generated by TXs.

Both TX and RX solenoids are connected to a capacitor in parallel, so as to have resonant LC circuits. In this realization of the MPS, we have $N_{tx}=36$, $S_{tx}=\pi 5^{2}$ mm${}^{2}$, $N_{rx}=252$, and $S_{rx}=\pi 9.5^{2}$ mm${}^{2}$. All solenoids are made with a 33 AWG (American Wire Gauge) enameled copper wire. An alternating voltage is supplied to each TX by a signal generator. RXs are disconnected from any external supply, with the only source of voltage being the electromotive force induced by the oscillating magnetic fields of each TX. Both TX and RX solenoids are shown in Figure 2, together with their respective circuit models.

#### 2.5.1. The TX Circuit

TX is supplied with voltage $V_{gen}$ generated by a waveform generator with an equivalent resistance $R_{eq}$. The impedance of the TX is easily calculated as $Z_{tx}^{-1}=$${\left(R+Z_{L}\right)}^{-1}+Z_{C}^{-1}=$${\left(R+i\omega L\right)}^{-1}+i\omega C$. The frequency response is Htxω=VtxVtxVgen=ZtxZtxZtx+ReqZtx+ReqVgen=ZtxZtxZtx+ReqZtx+Req, with the following amplitude:

(6) $${\left|H\left(\omega \right)\right|}^{2}=\frac{R^{2}+\omega^{2}L^{2}}{{\left[R_{eq}\left(1-\frac{\omega^{2}}{\omega_{0}^{2}}\right)+R\right]}^{2}+\omega^{2}{\left(R_{eq}RC+L\right)}^{2}}\underset{R\to 0}{=}\frac{\omega^{2}L^{2}}{{\left[R_{eq}\left(1-\frac{\omega^{2}}{\omega_{0}^{2}}\right)\right]}^{2}+\omega^{2}L^{2}},$$

where $\omega_{0}={\left(LC\right)}^{-\frac{1}{2}}$ is the resonance frequency when R=0. If *R* is slightly larger than 0, the shift of the resonance frequency from $\omega_{0}$ is negligible. Both $V_{tx}$ and $V_{gen}$ are directly measurable at different frequencies to obtain experimental values of $\left|H_{tx}\left(\omega \right)\right|$.

#### 2.5.2. The RX Circuit

In the circuit model of an RX, there is no external supply. A voltage $V_{\Phi }$ is induced across the coil by an external oscillating magnetic flux $\Phi =\Phi_{0}\sin\left(\omega t+\phi \right)$. By Faraday’s principle, $V_{\Phi }=-\frac{d\Phi }{dt}=-\omega \Phi_{0}\cos\left(\omega t+\phi \right)$. Thus, the following applies to the RX circuit:

(7) $$\begin{array}{cc} V_{R}+V_{C}-V_{L} & =V_{\Phi }\\ RI+\frac{Q}{C}+L\frac{dI}{dt} & =-\omega \Phi_{0}\cos\left(\omega t+\phi \right), \end{array}$$

with $I=\frac{dQ}{dt}$. Substituting *ansatz* QQCC=VC=VC0sinωt into the equation, the frequency response is easily obtained:

(8) $${\left|H_{rx}\left(\omega \right)\right|}^{2}={\left(\frac{V_{C0}}{\omega \Phi_{0}}\right)}^{2}=\frac{\omega_{0}^{4}L^{2}}{L^{2}{\left(\omega^{2}-\omega_{0}^{2}\right)}^{2}+\omega^{2}R^{2}}$$

If the capacitor is removed, leaving the circuit open, i.e., C=0, then $\left|H_{rx}\left(\omega \right)\right|=1\Rightarrow V_{C0}=\omega \Phi_{0}$, i.e., the voltage measured across the inductor is equal to the derivative of the magnetic flux, as expected by Faraday’s principle. It is possible to measure $\left|H_{rx}\left(\omega \right)\right|$ keeping a TX and an RX at fixed positions for a set of ω values; a direct measure of the voltage across the RX solenoid *without* a capacitor gives $\omega \Phi_{0}$, while measuring the same voltage *with* the capacitor gives $V_{C0}$. Their ratio is the experimental measurement of $\left|H_{rx}\left(\omega \right)\right|$.

#### 2.5.3. System Frequency Response

**Figure 3:**
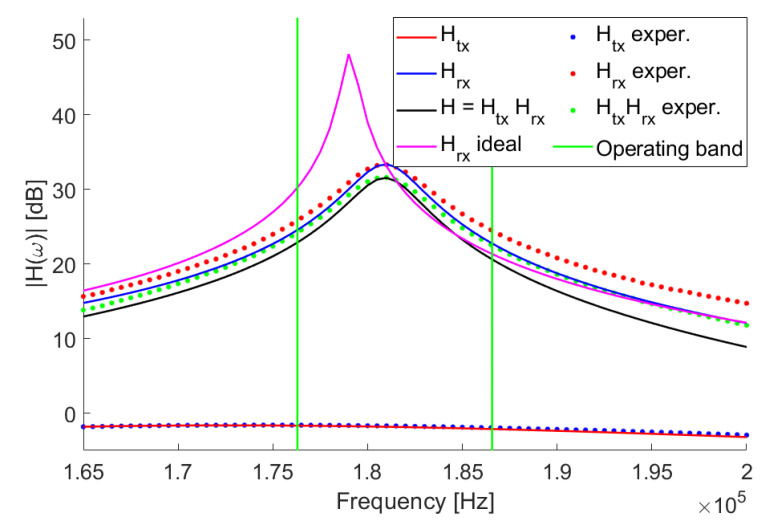
The frequency response, where solid lines are theoretical and dotted lines are experimental: the operating band (see Table 1) is delimited by the vertical green lines.

A LCR (inductor-capacitor-resistance) meter at 200 kHz was used to measure *C* and *L*. The measured values of resistance, capacitance, and inductance of the TXs are $R_{eq}=50\;\Omega$, R=1Ω, C=50 nF, and L=16.5μH, and that for RXs are R=6Ω, C=676 pF, and L=1150μH. When the voltages are measured using the oscilloscope, the input parasitic capacitance of the instrument (≈10 pF in our case) has to be added to *C*. The overall frequency response of the system can be estimated as $H\left(\omega \right)=H_{tx}\left(\omega \right)H_{rx}\left(\omega \right)$. Theoretical responses calculated using these parameters are shown in Figure 3, together with their experimental values for a TX and an RX. The theoretical curve for TX clearly matches the experimental one, while the theoretical curve for RX (the one indicated with “ideal” in the plot) is appreciably different from experimental values. This is due to the parasitic capacitance of the inductor and to the increase of resistance due to the skin and proximity effects as the frequency is increased. The varying magnetic field generated by the AC current induces eddy currents which cancel the current flow inside the conductor and concentrate the flow only in a layer near the surface; the depth of this layer is called skin depth. The proximity effect is instead due to eddy currents induced on a conductor winding by the varying magnetic field of the nearby windings. By including these effects as explained in the Appendix A, we obtained a much better theoretical curve (solid blue line in Figure 3).

#### 2.5.4. Driving the TX

**Figure 4:**
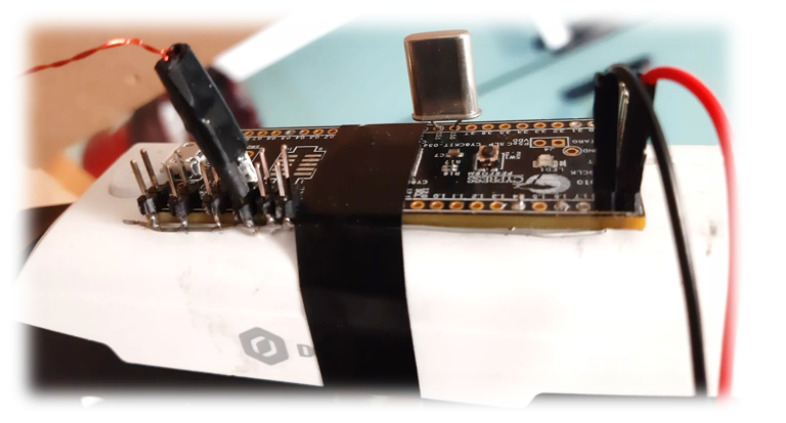
A PSoC5 microcontroller mounted on a robotic arm: connectors for the TXs are shown on the left. Only one TX is connected in this photo.

While designing the system, it is important to choose the frequencies of the signals supplied to each TX in order to match the high-response band of *H*$\left(\omega \right)$. Because of the device parameter tolerance, the actual responses of the solenoids are different. It is important to verify that the experimental response curves are largely superimposed. The chosen operating frequency band is delimited in Figure 3 by the green vertical lines. The alternating voltage is supplied to each TX through a square wave generated with a Cypress PSoC 5LP microcontroller (CY8CKIT-059 development board, Figure 4). The square wave has been used because it is easy to generate using the digital outputs (GPIO) of the PSoC. Each TX is connected to a different GPIO. The square wave component at fundamental frequency is selected by the narrow bandwidth of $H\left(\omega \right)$. The PSoC5 operates at low voltages (1.7 to 5.5 V); hence, it is suitable to be used with an autonomous power supply (e.g., a battery) as wearable electronics (e.g., data gloves). The amplitude of the square wave is controlled by regulating the voltage used to power the PSoC5. When several TXs are used instead of a single node, the signal received by each RX is the sum of the voltages induced by all TXs. Since, between each RX and the ADCs, an amplification stage is applied to measure small signals, this can cause saturation of the ADCs, in which the full scale is 3 V, when the TXs are too close to an RX. The solution we adopted is lowering the power voltage as the number of TXs increases. TX operating frequencies and PSoC5 power voltages are reported in Table 1; frequency values have been chosen to be as regularly spaced as possible given the clock divider values available on the PSoC5.

### 2.6. Functional Scheme

**Figure 5:**
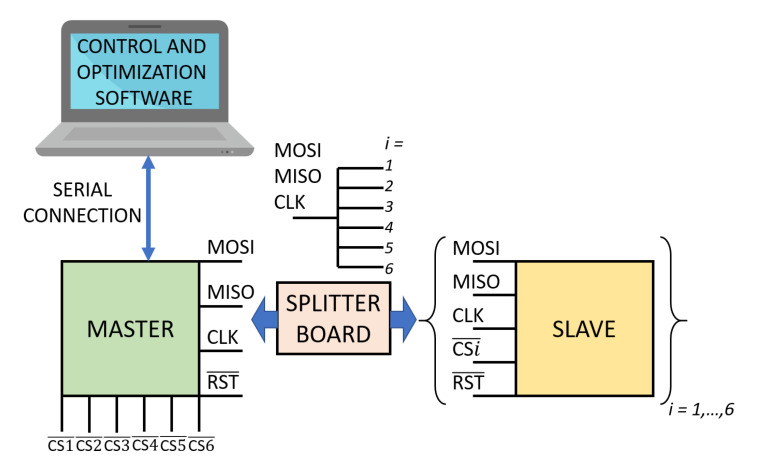
The basic functional scheme of the system: signal acquisition from RXs is performed by a network of slave microcontroller units (MCUs). Slaves are controlled by a central master MCU that triggers data acquisition on all slaves and reads all acquired data. Master–slave communication is based on an Serial Peripheral Interface (SPI) bus with star topology. Data are read from the master using a control software running on a standard PC; the same software uses the data to solve the optimization problem and to estimate position and attitude of all TXs.

The system is based on two main functional blocks, shown in Figure 5: the distributed DAQ, and the control and optimization software. The distributed DAQ is based on a network of MCUs communicating through a *Serial Peripheral Interface* (SPI), based on a master–slave architecture. We used a star configuration: the master-out-slave-in (MOSI), master-in-slave-out (MISO), and clock (CLK) lines from the master are split into six lines each, with each branch connected to a slave. There are six slaves. Each slave acquires signals on four RXs and performs the Fourier analysis to estimate voltage amplitudes and phases. The master triggers data acquisition on all slaves simultaneously and then collects all acquired data. There is also a *Reset* line (RST) to restart all slaves by operating only on the master MCU.The control and optimization software runs on a standard personal computer. This application communicates with the master MCU through a serial connection via the USB port. It triggers the master to start acquisition and then reads all voltage amplitudes and phases stored on the master. These data are used by the optimization routine to estimate the position and attitude of all TXs.

At the hardware level, the MOSI, MISO, and CLK lines of the SPI interface should be split from the master towards all slaves using a star topology. A short circuit simply made with cables and connectors could be the easiest solution, but we verified that it was not possible to connect more than two slaves. The fan-out of SPI ports on the Texas Instrument (TI) MCUs is not suitable to drive all the slaves, resulting in corrupted and practically unusable SPI signals. To solve this problem, we devised a custom splitter board using suitable bus drivers.

**Figure 6:**
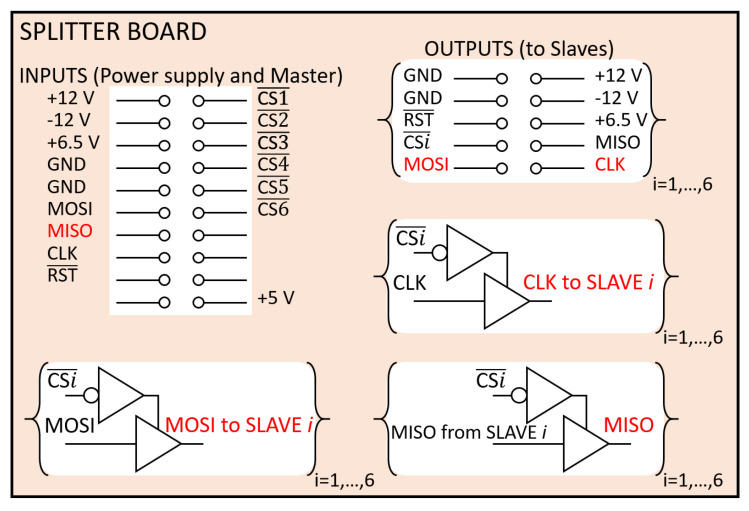
Simplified schematic of the splitter board with the bus drivers used to split SPI lines. The color red indicates buffered signals.

The splitter board (Figure 6) has an input connector for power supplies and master SPI ports and six output connectors for the slaves. Power supply input pins are simply short-circuited with the corresponding output pins.

We mounted three-state buffers on the board. We chose TI SN74ABT125 bus drivers since their maximum output current is 32 mA (one of the highest values we found on the market) and their switching time is very short (≈5 ns satisfies the requirements if one considers that our SPI CLK frequency is 3.125 MHz). The output enabled by these buffers is driven by $\bar{\mathrm{CS}}$ lines of the SPI. The MOSI line from the master is short-circuited to the inputs of six buffers, while each $\bar{\mathrm{CS}}$ line is used to enable only one buffer at a time. Each buffered output is connected to a different slave. The same scheme has been implemented for the CLK line from the master. In this scheme, each slave is driven exactly by one buffer; hence, the fan-out is not a problem anymore. The scheme is reversed for the MISO lines. The six MISO lines from each of the slaves are connected to the inputs of six buffers, while their outputs are all short-circuited and connected to the MISO pin of the master. In Figure 6, all lines driven by a buffer are indicated in red. The 5 V pin on the input connector is used to power the buffers.

### 2.7. Control and Optimization Software

The control software has two distinct modes: free hand motion and robot motion; when in the first mode, all TXs are moved freely by an operator and the software estimates their positions. When operating in the second mode, the software also controls a robotic arm to move the TXs along predefined trajectories; at each acquisition, the actual positions are read from the robot internal MCU, thus allowing to compare the positions estimated by the MPS with the nominal or groundtruth ones. This can be done only after the robot has been calibrated using the reference grid, as explained in [12]. The used robotic arm is a Dobot Magician by Shenzhen Yuejiang Technology. Being made of aluminium and acrylonitrile butadiene styrene (ABS) polymer, it is not ferromagnetic, and in [12], we verified the it does not produce any measurable distortion of magnetic fields. The Dobot is controlled through a dedicated application programming interface (API) [33].

**Figure 7:**
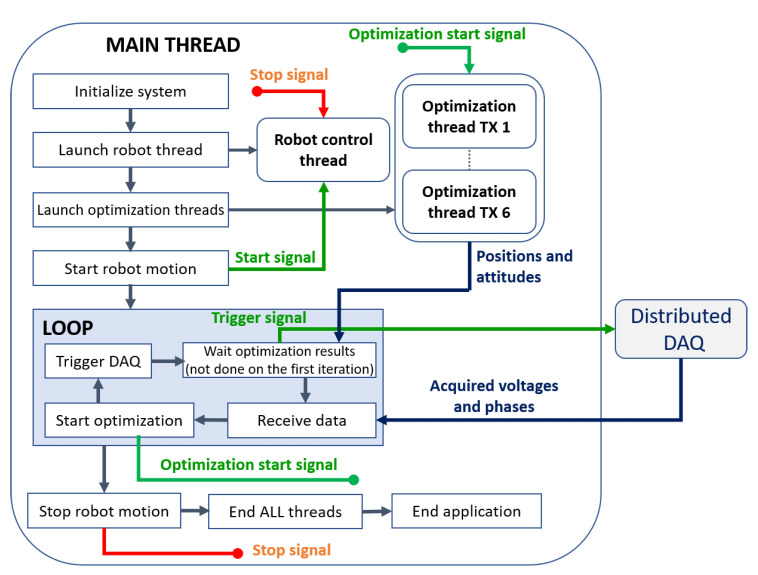
Simplified flow chart of the control software.

The control software is entirely developed in C++. A simplified flow chart of the software is shown in Figure 7. The robot is controlled by a dedicated thread running independently in order to move the robot and acquire data simultaneously. A predefined set of trajectory points is loaded, the robot thread is started, and then the program enters into its main loop. Through the serial port, the master MCU is triggered (which in turn triggers all slaves), and the application waits until data acquisition has finished and then reads all the voltage amplitudes and phases from the master. The optimization problem is solved by up to six concurrent threads (one for each TX). As in [12], we verified that six concurrent threads are faster than solving six optimization problems sequentially. The next data acquisition on the distributed MCU network is triggered immediately after the optimization threads have been started to perform concurrently both computation and acquisition for faster performances. Once the trajectory is completed (or the acquisition is stopped manually when running in the free hand mode), the main loop terminates. We tested the application on a computer equipped with a six-core Intel i7-8750H CPU at 2.20 GHz, running Windows 10.

### 2.8. FDMA and Band-Pass Sampling

**Figure 8:**
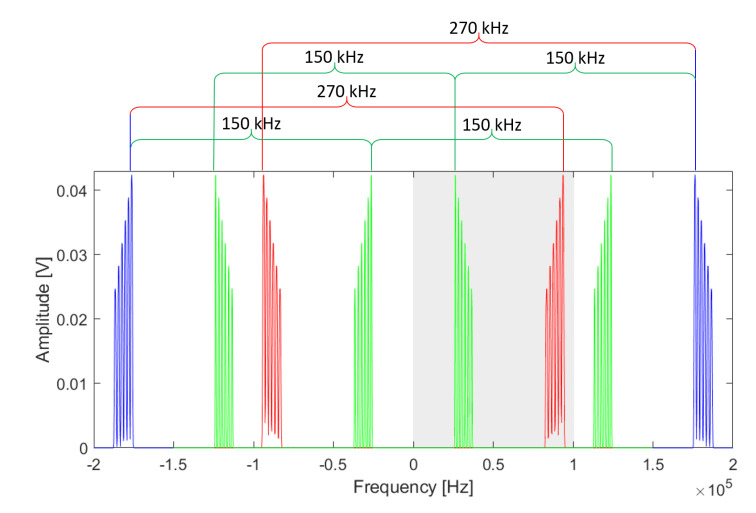
A visualization of band-pass sampling, where blue is the original signal spectrum $X\left(f\right)$, and red and green are the undersampled signal spectrum $X_{S}\left(f\right)$ in which red is $f_{S}=270$ kHz (m=1) and green is $f_{S}=150$ kHz (m=2): the discrete Fourier transform (DFT) returns only the spectral portion within the shadowed area of the plot.

As explained in Section 2.5, each TX solenoid is supplied with a different voltage signal frequency. The operating band is centered aroung $f_{c}=182$ kHz, and the bandwidth is B=10 kHz. The separation between two near TX frequencies is about 2 kHz. In order to discriminate two near frequency components, the frequency resolution of the acquisition system has to be a few hundred hertz or better. The frequency resolution of the discrete Fourier transform (DFT) is Δf=fSfSNSNS, where $f_{S}$ is the sampling frequency and $N_{S}$ is the number of samples. Acquiring at Nyquist rate ($f_{S}\geq 2\times 182$ kHz) would imply the acquisition of 2048 or more samples. Each MCU acquires signals on four RXs simultaneously, and 4×2048 samples exceed its memory capacity. In order to reduce $N_{S}$, $f_{S}$ has to also be reduced below the Nyquist frequency. Since the bandwidth of TX signals is narrow, it is possible to apply the band-pass sampling technique [34]. If $X\left(f\right)$ is the Fourier transform of a signal $x\left(t\right)$, then the Fourier transform of the signal sampled with sampling rate fS=11TSTS is

(9) $$x\left(nT_{S}\right)\Leftrightarrow X_{S}\left(f\right)=\sum_{k=-\infty }^{+\infty }X\left(f-kf_{S}\right),$$

i.e., $X_{S}\left(f\right)$ is the periodic repetition, with period $f_{S}$, of $X\left(f\right)$. In Figure 8, the original spectrum for six TXs (blue) at arbitrary positions and a couple of undersampled signal spectra, with $f_{S}=270$ kHz (red) and $f_{S}=150$ kHz (green), are shown. It is important to choose $f_{S}$ such that spectral repetitions do not overlap. The nonoverlapping conditions are as follows:

(10) $$\begin{array}{cccc} f_{S^{\prime \prime }} & \leq f_{S}\leq f_{S^{\prime }}, & f_{S} & \geq 2B \end{array}$$

(11) $$\begin{array}{cccc} f_{S^{\prime }} & =\frac{2f_{C}-B}{m},\; & f_{S^{\prime \prime }} & =\frac{2f_{C}+B}{m+1}, \end{array}$$

where *m* is an arbitrary integer indicating how many spectral repetitions there are in the interval $\left[-f_{c},f_{c}\right]$, i.e., within the original spectrum. The optimal sampling frequency is the mean fS=fS′+fS″fS′′+fS′′22. In Figure 8, we used m=1 (red) and m=2 (green). The DFT returns only the part of the spectrum within the shadowed area (i.e., a single spectral repetion); hence, when using an odd *m*, the spectrum is flipped and care must be taken in order to correctly associate each peak with its correspondig TX. We chose $f_{S}=270$ kHz (m=1) and $N_{S}=1024\Rightarrow \Delta f=264$ Hz. The memory capacity of MCUs supports $N_{S}=1024$.

A noticeable difference of the present MPS compared to the DAQ system devised in [29] is that no fitting of the acquired signal is performed. Only the amplitude and phase of the signal are needed in the computation, and they are directly obtained from the spectrum calculated with the Fast Fourier Transform algorithm. Flat-top windowing is applied to the acquired data, optimized to give minimal scalloping loss [35].

### 2.9. Microcontroller Programming

Following the analysis in [29], we built the distributed DAQ architecture using Texas Instruments (TI) Delfino TMS320F28379D [36] microcontrollers, mounted on the TI LaunchXL development board. TI Delfino has a 200 MHz dual-core CPU and four 12-bit ADCs with a maximum sampling rate of 3.5 MSa/s. The MCU can be programmed using C++ and a dedicated compiler provided by TI. There are also two control law accelerators (CLA), i.e., additional co-processors that can execute simple operations in parallel with the main CPU. Other features relevant for our purposes are the direct memory access module (DMA), two SPI interfaces, and two serial communication interfaces (SCI; known as Universal Asynchronous Receiver-Transmitter (UART)). We used one SCI to connect the master MCU with the personal computer. The TI Delfino includes a floating point unit (FPU) co-processor for each core, supporting 32-bit single-precision floating point operations, that we used with the specific mathematical library provided by TI implementing a device-optimized FFT algorithm [37]; an interprocessor communication (IPC) module for communications between the two cores, that we used to control parallel operations; and a pulse width modulator (PWM), that we used to generate a square wave applied to trigger the ADCs at fixed sampling frequency.

**Figure 9:**
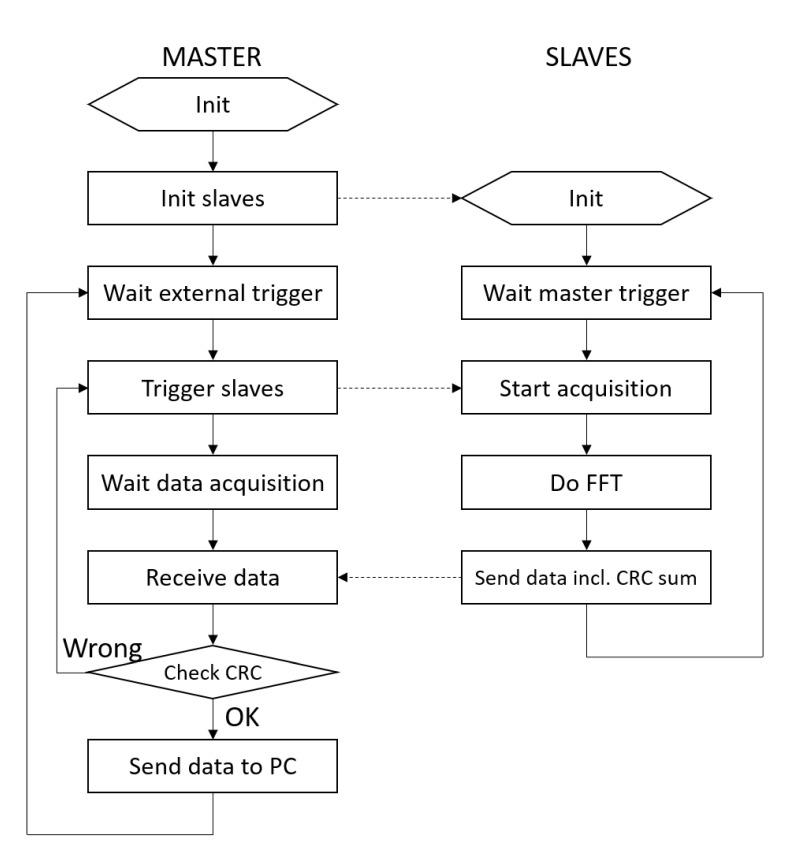
Simplified flow chart of master and slave operations.

Figure 9 shows a simplified flow chart of both master and slaves operations. Once started, the master sends a set of configuration parameters (sampling frequency, number of TXs, TXs frequencies, and the bandpass parameter *m*) through SPI to all slaves to avoid reprogramming each slave whenever one needs to use different configurations. When the init phase has completed, the master enters a wait state until it receives an external trigger from the computer through the SCI. Immediately after, the master triggers all slaves simultaneously. Each slave uses its four independent ADCs to acquire signals on each RX. A DFT-based spectrum estimation is obtained for each RX. The maxima of its magnitude are found, and amplitudes and phases are stored in a vector. A cyclic redundancy check (CRC) [38] is applied, and the checksum is appended to the vector. After 6 ms (the fixed duration of signal acquisition and processing), the master reads through SPI the resulting vector from each slave and recalculates the checksum to verify that data have not been corrupted during transfer. If the checksum is correct, a time reference in μs, to be used in the Kalman Filter, is read from an internal timer of the master MCU. All data are sent to the computer through the SCI, and finally, the master returns in its initial state, waiting for an external trigger.

#### 2.9.1. SPI Communication

SPI communication is controlled by the master. Slaves are programmed as finite-state machines; a global variable is used to define the state. On a slave, SPI communication is handled by a CLA to allow communication without interrupting the operations of the main CPU. When a word is received through the SPI, the CLA checks the state variable, executes the operations associated with the current state, and in case, changes the state.

In the initial state, the slave assumes the received words as configuration parameters and stores them in proper variables. As the last parameter has been received, the slave switches to the ready state and it can accept a trigger from the master. As signal acquisition has to be started simultaneously on all RXs, the master sends a trigger signal to all slaves at the same time, lowering all $\bar{\mathrm{CS}}$ lines simultaneously. When triggered, a slave switches to the busy state and starts signal acquisition and processing. As all data are ready to be transferred, the slave switches to the data_ready state. In this phase, for any dummy word sent by the master, the slaves writes a data word on the MISO line until all data are transferred; then, the slave switches back to its ready state.

#### 2.9.2. Signal Acquisition and Processing

As a slave is triggered, the PWM, by writing on the proper control register, is internally connected to the ADC’s interrupt line to trigger signal acquisition at fixed sampling frequency $f_{S}$. As a sample is acquired and converted, its value is written on the ADC result buffer and an interrupt signal is sent to the DMA that reads this buffer and writes the value on a vector cell, automatically increasing a pointer to the next cell for the subsequent sample. Once $N_{S}$ samples have been acquired, the PWM trigger is disconnected, the DMA pointer is reset, and the acquisition phase finishes.

There are four data vectors, one for each RX. The following sequence of operations is executed on each vector: the flat-top window function is applied to the data; the FFT is calculated; and the maxima of the magnitude spectrum are found: since TX frequencies are known *a priori*, maxima are directly found around their nominal values; hence peak finding is very fast. Since the CPU is dual-core, two sequences of operations are executed in parallel, halving the execution time. According to the FPU library documentation, for $N_{S}=1024$, the execution time of the FFT algorithm is 152 μs.

### 2.10. Slave Boards

**Figure 10:**
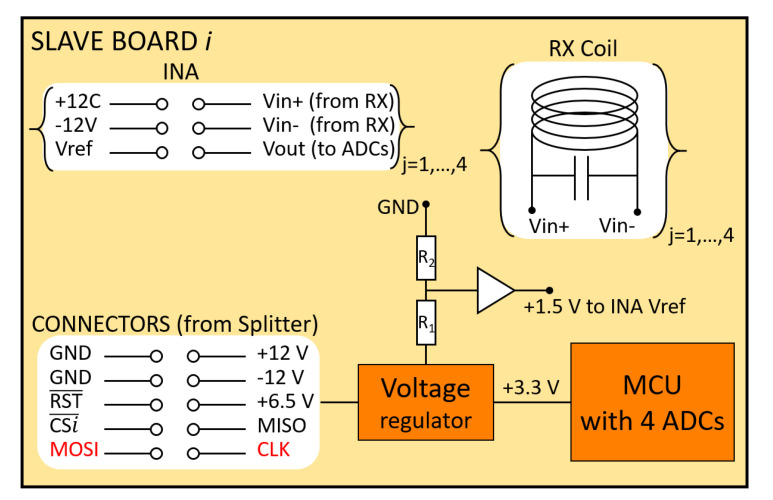
Simplified schematic of a slave board: the color red indicates buffered signals.

The slave circuit is shown in Figure 10. Each slave MCU is connected to four RX solenoids. The signal of each RX has to be amplified to match the ADC input range. We implemented all these parts on a custom circuit board, one for each slave. The input connector of each board is wired to an output connector of the splitter board. A voltage regulator is mounted on the slave board, powered through the 6.5 V pin, and used to power the MCU with 3.3 V. The value 6.5 V from an external supply was chosen to compensate a slight voltage drop along the wires and the drop-out of the regulator. Each RX is connected to the inputs of an AD8421 instrumentation amplifier (INA) for which the nominal gain is set to 8. There are four INAs powered through the ±12 V connectors. Since the output signal of an RX is alternating, while the ADCs are monopolar, an offset has to be added to the signal to make it oscillate between 0 and 3 V, that is, the ADC voltage range. The offset is added through the $V_{ref}$ pin of the INA. It is obtained with a resistive divider connected to the output of the voltage regulator and connected to $V_{ref}$ through an op-amp buffer.

## 3. Testing the Magnetic Positioning System

To test the performance of the MPS, we set up six calibration and measurement stages, from a single TX to six TXs. For short terminology, we call one-node (or one-TX) configurations (or systems) an MPS tracking a single TX node and two-node, three-node, etc. configurations, an MPS tracking respectively two, three, etc. TX nodes simultaneously. Since, in different configurations, the TXs are supplied with different voltages and there can be mutual induction effects too, parameters resulting from the calibration in a particular configuration cannot be used with other configurations. A calibration procedure has to be performed for each configuration. The calibrated MPS is then used to track a reference trajectory in order to estimate the position and attitude errors. In Section 3.1, we describe the calibration and measurement operations, and in Section 3.2, the results will be presented.

### 3.1. Calibration and Measurement

**Figure 11:**
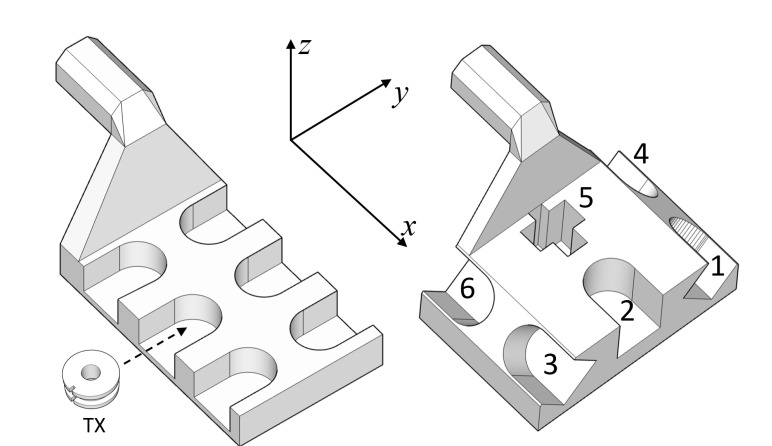
TX node supports for calibration (**left**) and measurement (**right**): the robot reference frame is reported. In this reference frame, the attitudes of the second support, using direction cosines notation, are ${\hat{n}}_{1}={\hat{n}}_{4}=\;\left(0,\left.\sqrt{2}/2\right.,\left.\sqrt{2}/2\right.\right)$, ${\hat{n}}_{3}={\hat{n}}_{6}=\;\left(0,\left.-\sqrt{2}/2\right.,\left.\sqrt{2}/2\right.\right)$, ${\hat{n}}_{2}=\;\left(0,0,1\right)$, and ${\hat{n}}_{5}=\;\left(1,0,0\right)$.

In order to operate with all TXs mounted on the robotic arm, we 3D-printed the two supports shown in Figure 11 to be used as end effectors of the robot, fixing all TXs in the designated slots. The first support was used for calibration, since it keeps all TXs at the same attitude along the *z* axis to avoid the calibration accuracy varying among the TXs because of different attitudes. Defining the attitude in terms of elevation and azimuth angle, the Dobot arm, by construction, keeps the elevation fixed while moving, and only the azimuth changes along the trajectory. However, when the attitude is directed along the *z* axis (elevation 90${}^{{}^{\circ}}$), it does not depend on the azimuth because of the cylindrical symmetry of this configuration.

For the measurement stage instead, we used the second support (attitudes are reported in the caption of Figure 11). As the robot moves, the elevations are kept constant at 90${}^{{}^{\circ}}$ (${\hat{n}}_{2}$), 0${}^{{}^{\circ}}$ (${\hat{n}}_{5}$), and 45${}^{{}^{\circ}}$ (${\hat{n}}_{1}$, ${\hat{n}}_{3}$, ${\hat{n}}_{4}$, and ${\hat{n}}_{6}$), while the azimuth changes along the trajectory (except of course for ${\hat{n}}_{2}$). With this support, we can estimate the position and angle accuracy of the MPS for three different elevations.

**Figure 12:**
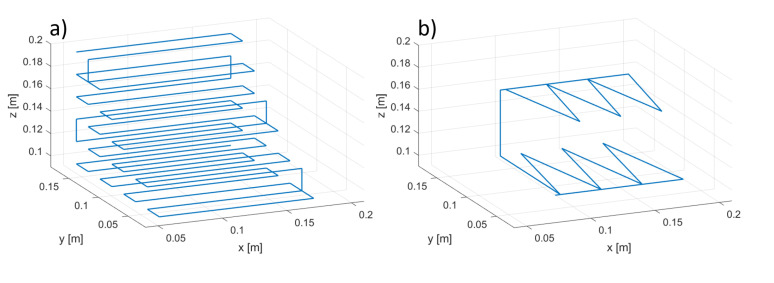
Calibration trajectory (**a**) and measurement trajectory (**b**).

Following the analysis of [12], the calibration trajectory was chosen to span as large a part as possible of the active volume of the MPS. The calibration trajectory is shown in Figure 12a. Of course, each TX on the support travels along a slightly different trajectory, and this figure shows only one out of six trajectories. The system is calibrated by minimizing the cost function (5), as explained in Section 2.3 and in [12]. All TXs are active while the signal is being acquired, as explained in Section 2.8 and Section 2.9.2. There is a minimization problem for each TX, each one independent from the others, i.e., different sets of ${\hat{\theta }}_{rx,i}$ and ${\hat{C}}_{rx,i}$ values are obtained for each TX.

After the calibration procedure was performed for all configurations, we proceeded with the measurement stages, mounting all TXs on the second support shown in Figure 11 and using the robot to move it along the trajectory of Figure 12b. Since the support has slots with different attitudes, we performed several measurements, testing each of the TXs on each and every slot to estimate the accuracy of each TX for different attitudes and positions. As an example, for the one-node configuration, we tracked a single TX six times, one for each slot; for the six-node configuration, we tracked six TXs six times, the first time mounting TX1–TX6 in slots 1–6; then in slots 2–6 and 1; then in slots 3–6, 1, and 2; and so on. The results are discussed in the next section.

### 3.2. Experimental Results

The first interesting result is that, although they are operated using different frequencies, the accuracy is practically the same for each TX, i.e., varying the frequency within the band defined in Section 2.8, with $f_{c}=182$ kHz and B=10 kHz, does not produce any appreciable difference in the accuracy of the system. All the results presented in the following are thus an average over all the TXs of each configuration (see Table 1). The measurement rates for different configurations are reported in Table 2.

**Figure 13:**
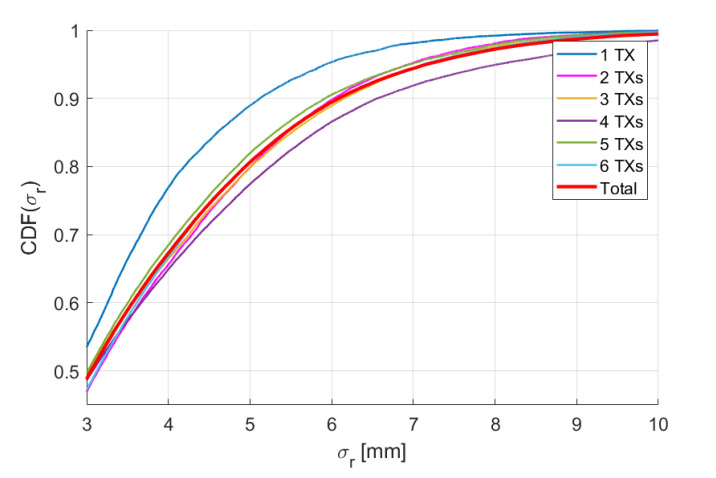
Cumulative distribution functions of $\sigma_{r}$.

**Figure 14:**
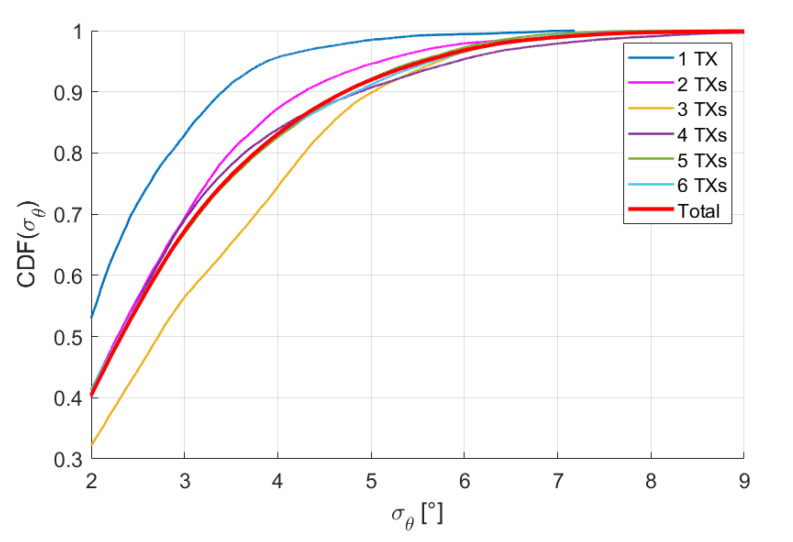
Cumulative distribution functions of $\sigma_{\theta }$.

We define the position accuracy $\sigma_{r}$ of the system as the Euclidean distance between the position measured with the MPS and the real position provided by the robot, while the angular accuracy $\sigma_{\theta }$ is defined as the angle between the measured attitude $\hat{n}$ and the real attitude n, i.e., $\sigma_{\theta }=\cos^{-1}\left(\frac{\hat{n}\cdot n}{\left|\hat{n}\right|\left|n\right|}\right)$. Measuring the accuracies for each trajectory point, we get distributions for both $\sigma_{r}$ and $\sigma_{\theta }$. The percentiles of $\sigma_{r}$ and $\sigma_{\theta }$ are reported in Table 3 and Table 4 for all configurations. The last row of both tables contains the percentiles calculated on the union of all distributions with respect to each configuration. The cumulative distribution functions (CDF) of $\sigma_{r}$ and $\sigma_{\theta }$ for each configuration and for the union distribution are reported in Figure 13 and Figure 14. The CDFs of different configurations are discernible, but the percentiles do not differ by more than ≈0.5 mm and ≈0.5${}^{{}^{\circ}}$, the accuracy of the system thus being practically the same for any configuration.

**Figure 15:**
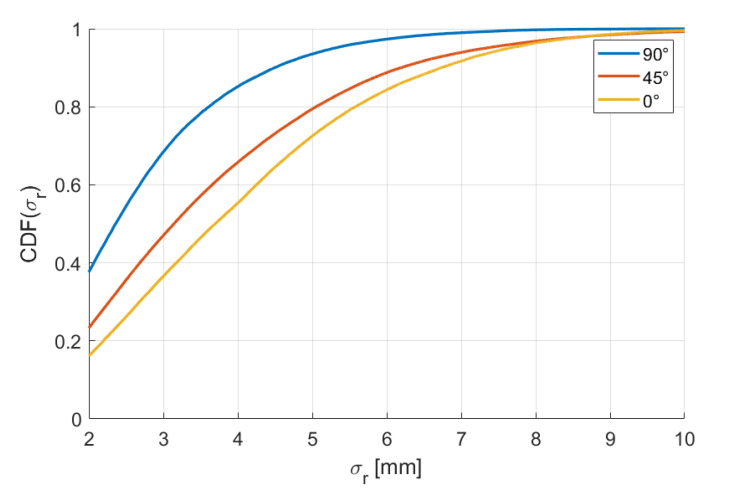
Cumulative distribution functions of $\sigma_{r}$ for elevations $90^{{}^{\circ}}$, $45^{{}^{\circ}}$, and $0^{{}^{\circ}}$.

**Figure 16:**
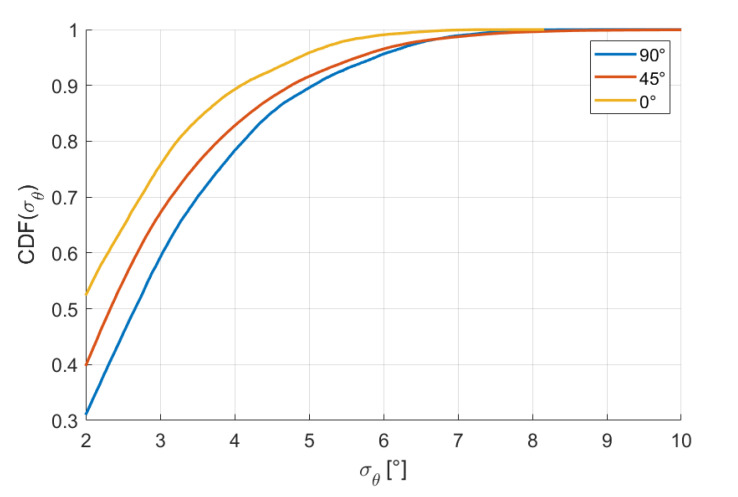
Cumulative distribution functions of $\sigma_{\theta }$ for elevations $90^{{}^{\circ}}$, $45^{{}^{\circ}}$, and $0^{{}^{\circ}}$.

Since the arrangement of the RXs is not symmetrical (16 RXs on the xy plane and 4 RXs on both the xz and yz planes), we examined if the accuracy has some dependence from the attitude elevation angle. In fact, it has the best position accuracy obtained for the vertical attitude ($90^{{}^{\circ}}$ elevation) and the worst position accuracy for the horizontal attitude ($0^{{}^{\circ}}$ elevation); opposite results are obtained for the angle accuracy, the best values being obtained for the horizontal attitude and the worst being obtained for the vertical attitude. The percentiles for elevations $90^{{}^{\circ}}$, $45^{{}^{\circ}}$, and $0^{{}^{\circ}}$ are reported in Table 5 and Table 6. The corresponding CDFs are reported in Figure 15 and Figure 16.

In order to understand the effect of mutual induction among the TXs and the effect of lowering the voltage supply as the number of TXs increases (see Section 2.5), we calculated the signal-to-noise ratio (SNR) for the 1-node configuration and the 6-nodes configuration. We define the signal as the ideal voltage $V_{rms}\left(k\right)$ calculated using (3) at each point of the reference trajectory traced by the robotic arm, while the noise is defined as the difference between the ideal and the measured voltages ${\tilde{V}}_{rms}\left(k\right)$:

(12) $$SNR_{dB}\left(k\right)\;=\;20\log\left(\left|\frac{V_{rms}\left(k\right)}{V_{rms}\left(k\right)-{\tilde{V}}_{rms}\left(k\right)}\right|\right).$$

**Figure 17:**
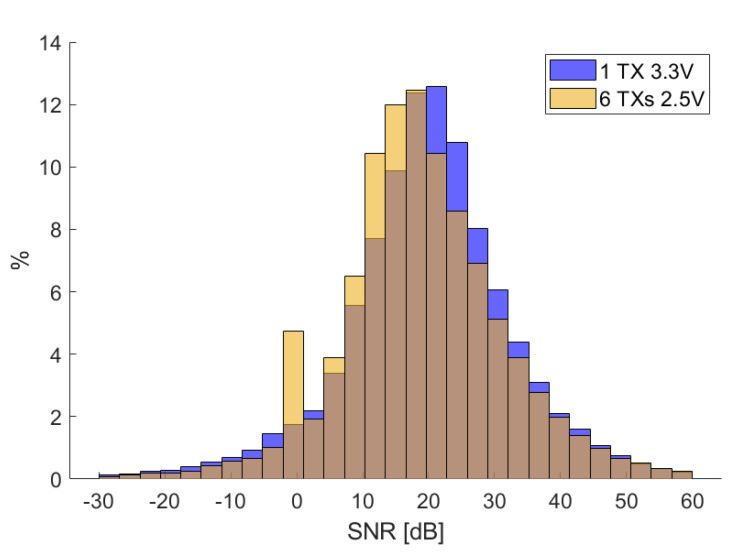
Signal-to-noise ratio (SNR) histogram obtained using (12) for all RXs.

In Figure 17, we report the two distributions, and in Table 7, we report their mean and standard deviation. The SNR is not so much affected by the voltage reduction and the mutual induction between nodes; this is consistent with the position and attitude errors that do not increase too much as the system is upscaled.

## 4. Conclusions

A magnetic positioning system capable of tracking the position and orientation of multiple coils in real time was presented and characterized. A median position accuracy of 3.1 mm and a median orientation accuracy of 2.4 degrees were observed with respect to reference measurements obtained using a robotic arm. The system can perform 62 measurements per second when tracking six nodes and up to 124 measurements per second when tracking one node only. The results show that the measurement accuracy of the system is very similar among different configurations. This is a clear hint that the system could be upscaled to more than six nodes. The most important limiting factor would be the saturation problem. Indeed, it is not possible to lower the transmitter supply voltage anymore because of the sensitivity limitations of the most distant RXs. This would result in a reduction of the effective number of receivers, compromising measurement accuracy. There are some possible solutions to overcome the saturation problem for future developments. One is to implement an automatic gain control on the INAs to dynamically reduce the gain as the signal amplitude approaches the saturation threshold. Another possibility is to increase the number of receivers, thus assembling them on a finer grid and then tuning different sets of TXs and RXs on different resonance bands. Lastly, ADCs with a greater range and a finer resolution could be used, e.g., 16-bit ADCs in place of 12-bit ADCs. This solution could not be implemented with the off-the-shelf components we used, since the 12-bit ADCs with fixed range are integrated in the microcontroller IC, but it is perfectly feasible in principle. 

## Figures and Tables

**Table 1:** Operating frequencies of TXs and PSoC power voltages.

TX Freqs [Hz]	Selected TXs					
176,296					X	X
178,259				X	X	X
180,266		X	X	X	X	X
182,319	X	X	X	X	X	X
184,420			X	X	X	X
186,569						X
PSoC supply [V]	3.3	3.3	3.3	2.7	2.7	2.5

**Table 2:** Measurement rates (sample/s) for all configurations.

Configuration:	1-TX	2-TXs	3-TXs	4-TXs	5-TXs	6-TXs
Meas. rate:	124	83	83	83	62	62

**Table 3:** Percentiles of $\sigma_{r}$ (mm) for all configurations.

	50%	75%	95%	99%	100%
**1 TX**	2.9	3.9	5.9	7.7	11.7
**2 TXs**	3.1	4.6	6.9	8.6	13.1
**3 TXs**	3.0	4.6	7.1	9.0	14.1
**4 TXs**	3.1	4.8	8.0	10.5	15.5
**5 TXs**	3.0	4.4	7.0	8.7	14.0
**6 TXs**	3.1	4.5	7.1	9.0	13.8
**Tot:**	3.1	4.5	7.2	9.3	15.5

**Table 4:** Percentiles of $\sigma_{\theta }$ (${}^{{}^{\circ}}$) for all configurations.

	50%	75%	95%	99%	100%
**1 TX**	1.9	2.6	3.8	5.4	7.2
**2 TXs**	2.3	3.2	5.1	7.1	9.0
**3 TXs**	2.7	4.0	5.7	7.1	9.9
**4 TXs**	2.3	3.3	5.9	7.9	11.4
**5 TXs**	2.3	3.4	5.5	6.6	8.6
**6 TXs**	2.3	3.4	5.6	6.9	9.2
**Tot:**	2.4	3.4	5.6	7.0	11.4

**Table 5:** Percentiles of $\sigma_{r}$ (mm) for elevations $90^{{}^{\circ}}$, $45^{{}^{\circ}}$, and $0^{{}^{\circ}}$.

	50%	75%	95%	99%	100%
**$90^{{}^{\circ}}$**	2.4	3.3	5.3	7.0	10.4
**$45^{{}^{\circ}}$**	3.1	4.7	7.3	9.5	15.5
**$0^{{}^{\circ}}$**	3.7	5.2	7.6	9.3	13.3

**Table 6:** Percentiles of $\sigma_{\theta }$ (${}^{{}^{\circ}}$) for elevations $90^{{}^{\circ}}$, $45^{{}^{\circ}}$, and $0^{{}^{\circ}}$.

	50%	75%	95%	99%	100%
**$90^{{}^{\circ}}$**	2.7	3.8	5.9	7.1	10.2
**$45^{{}^{\circ}}$**	2.3	3.4	5.6	7.2	11.4
**$0^{{}^{\circ}}$**	1.9	3.0	4.9	6.0	8.2

**Table 7:** SNR mean and standard deviation.

	1 TX (3.3 V)	6 TX (2.5 V)
${\hat{\mu }}_{snr}$ (dB)	20.0	18.9
${\hat{\sigma }}_{snr}$ (dB)	13.4	13.1

**Figure A1:**
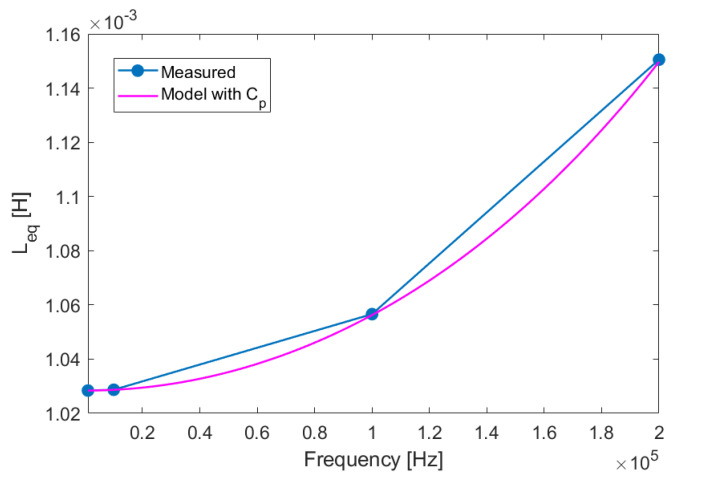
Measured inductance of an RX coil (blue) and its model given by Equation (A1) (magenta).

**Figure A2:**
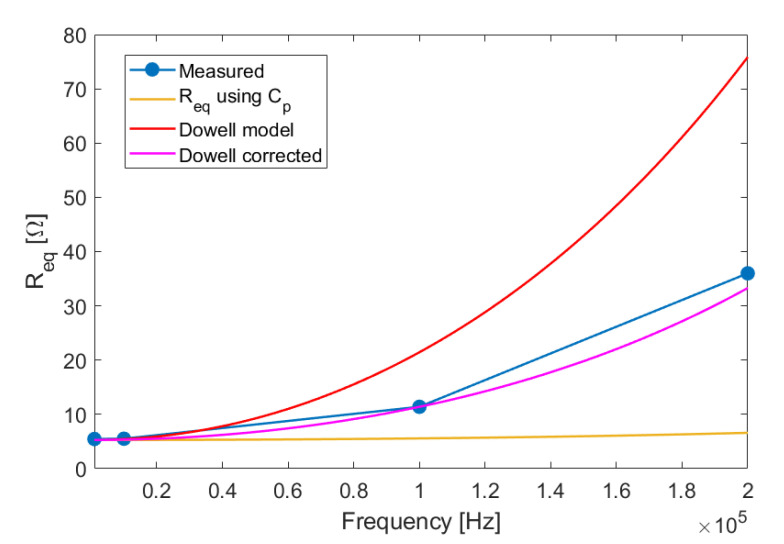
Blue represents the measured resistance of an RX coil. Yellow represents $R_{eq}$ given by Equation (A2). Red represents $R_{eq}$ given by the Dowell model accounting for the skin and proximity effects, using Equation (A3). Magenta represents the Dowell model with corrected porosity factor.

## Appendix A. Inductor and Resistance Models for RX Solenoids

Since each RX coil has 252 closely packed windings of enameled wire arranged in 6 layers, it has an intrinsic parasitic capacitance $C_{p}$ that has to be taken into account. The parasitic capacitance is modeled adding a capacitor in parallel to the R−L series of Figure 2 (bottom). An equivalent impedance $Z_{eq}$ is obtained as $Z_{eq}^{-1}={\left(R+i\omega L\right)}^{-1}+i\omega C_{p}$, that can be written using an equivalent resistance $R_{eq}$ and inductance $L_{eq}$ as $Z_{eq}=R_{eq}+i\omega L_{eq}$:

(A1) $$\begin{array}{c} L_{eq}=\frac{L-\omega^{2}L^{2}C_{p}-R^{2}C_{p}}{{\left(1-\omega^{2}LC_{p}\right)}^{2}+\omega^{2}R^{2}C_{p}^{2}} \end{array}$$

(A2) $$\begin{array}{c} R_{eq}=\frac{R}{{\left(1-\omega^{2}LC_{p}\right)}^{2}+\omega^{2}R^{2}C_{p}^{2}}. \end{array}$$

Using a bench LCR meter, we measured both $L_{eq}$ and $R_{eq}$ at 1, 10, 100, and 200 kHz, obtaining the values reported in Figure A1 and Figure A2. The low-frequency values have to be used for *L* and *R*. According to (A1), experimental $L_{eq}$ can be fitted to obtain $C_{p}=65$ pF (Figure A1, magenta). Using this value of $C_{p}$ to calculate $R_{eq}$ according to (A2), we obtain the yellow curve of Figure A2, which clearly does not match the experimental values.

In order to correctly model $R_{eq}$ in the AC regime, one has to take into account the skin effect and the proximity effect. In the first case, the varying magnetic field generated by the AC current induces eddy currents which cancel the current flow inside the conductor and concentrate the flow only in a layer near the surface; the depth of this layer, called skin depth, is given by δω=2ρ2ρμωμω, where ρ is the resistivity of the conductor and μ is its magnetic permeability; the second effect is instead due to eddy currents induced on a conductor winding by the varying magnetic field of the nearby windings. We can define the ratio FR=RACRACRDCRDC of the AC resistance including skin and proximity effects over the DC resistance. An analytical model for $F_{R}$ was derived by Dowell [39]:

(A3) $$F_{R}=A\left[\frac{\sinh\left(2A\right)+\sin\left(2A\right)}{\cosh\left(2A\right)-\cos\left(2A\right)}+\frac{2\left(N_{l}^{2}-1\right)}{3}\frac{\sinh\left(A\right)-\sin\left(A\right)}{\cosh\left(A\right)+\cos\left(A\right)}\right],$$

where $A={\left(\frac{\pi }{4}\right)}^{\frac{3}{4}}\frac{d}{\delta_{\omega }}\sqrt{\eta }$; *d* is the wire diameter; η=ddpp is the so-called porosity factor, accounting for the space between the windings; *p* is the distance between the centers of the adjacent winding conductors; and $N_{l}$ is the number of windings layers. The first term of the sum on the right-hand side of (A3) accounts for the skin effect, while the second term accounts for the proximity effect. Applying the Dowell model, i.e., substituting *R* with $F_{R}R$ in (A2), we obtained the red curve in Figure A2, which clearly overestimates the resistance of the inductor at higher frequencies. This behavior is due to the proximity term and in particular to the definition of the porosity factor. This is a known limit of the Dowell model, and usually, the porosity factor has to be fitted from finite elements simulations for different coil geometries, as in [40,41]. In the proximity term of (A3), we just treated η as a parameter that we determined by fitting the experimental resistance of Figure A2, thus obtaining a good model of $R_{eq}$ (magenta curve in Figure A2). We verified that the equivalent inductance $L_{eq}$ is not appreciably influenced by skin and proximity effects. Substituting *R* and *L* with $R_{eq}\left(\omega \right)$ and $L_{eq}\left(\omega \right)$ into (8), we obtained a theoretical response curve very similar to the measured one (blue curve in Figure 3).
